# Supplementary material for: The augmin complex architecture reveals structural insights into microtubule branching
Source: Nat Commun. 2022 Sep 26;13:5635. doi: 10.1038/s41467-022-33228-6 (PMC9512787; doi:10.1038/s41467-022-33228-6)
Supplement: Supplementary file 1 — Supplementary Information [file 41467_2022_33228_MOESM1_ESM.pdf]

Supplementary Information

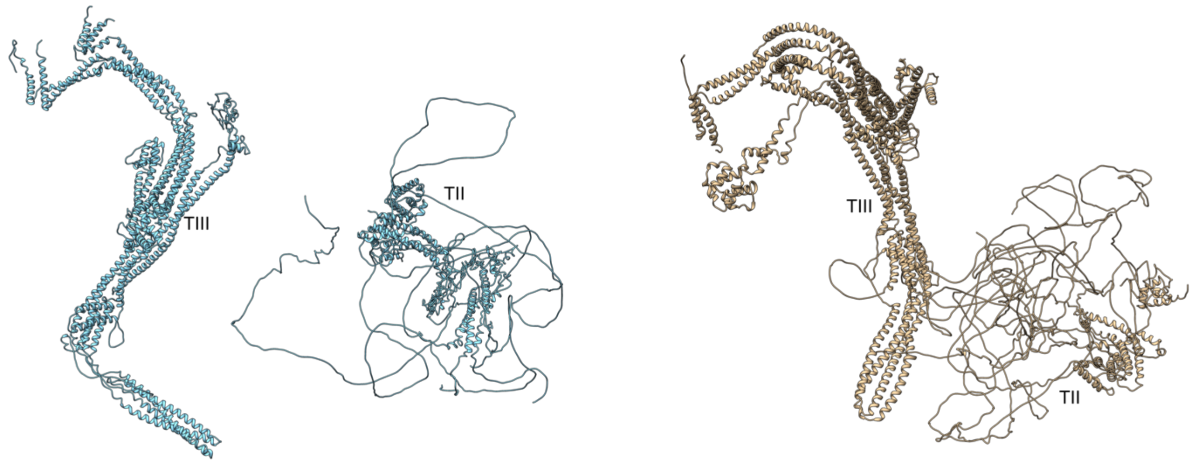

Supplementary Fig. 1: Prediction of the augmin holocomplex structure fails. The two highest-scoring models of the augmin holocomplex predicted by AlphaFold-Multimer. TIII and TII tetramers are labelled in both models.

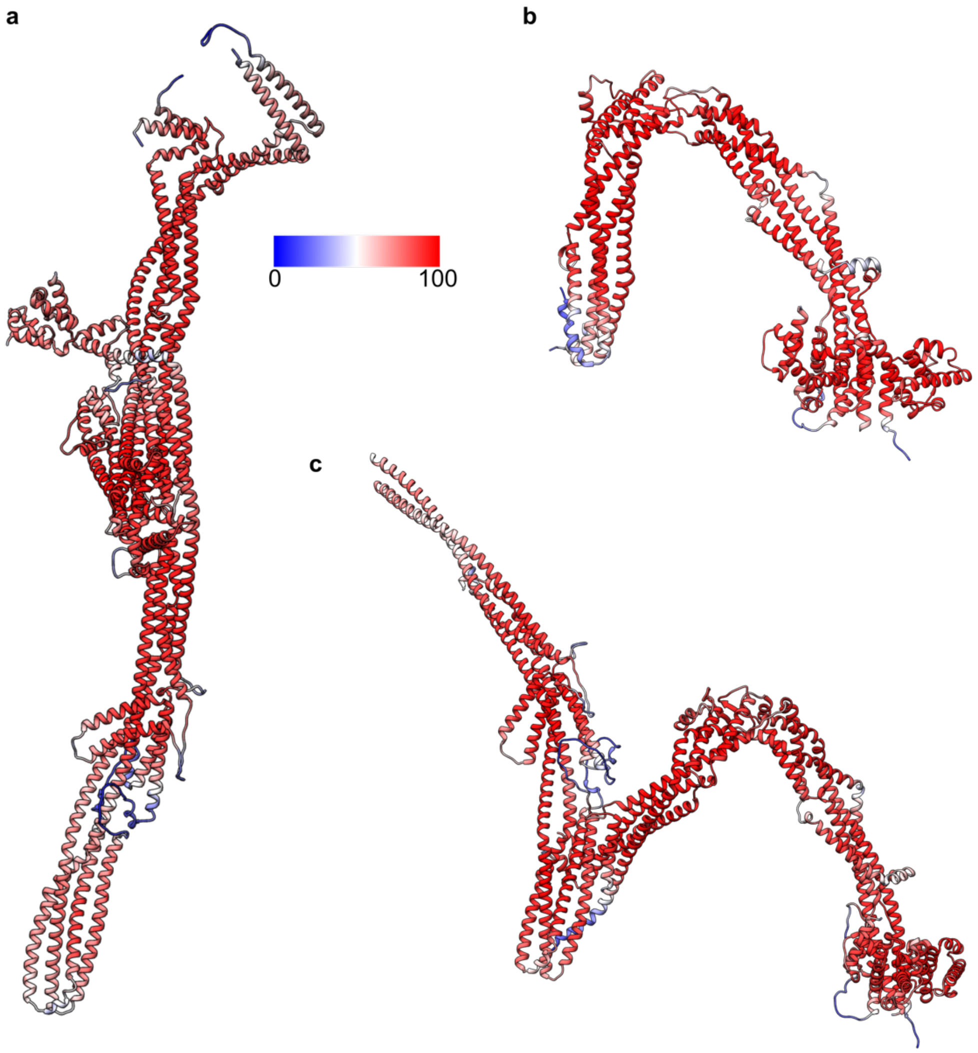

Supplementary Fig. 2: Per-residue confidence metric for the predicted models. a The highest-scoring augmin TIII tetramer model coloured according to pLDDT score. Colouring scheme and score scale is given. **b** The highest-scoring augmin TII tetramer model coloured according to pLDDT score. Scheme and scale same as in (**a**)**. c** The highest-scoring model for the TIII H3/H5-arm plus TII coloured according to pLDDT score. Scheme and scale same as in (**a**)**.**

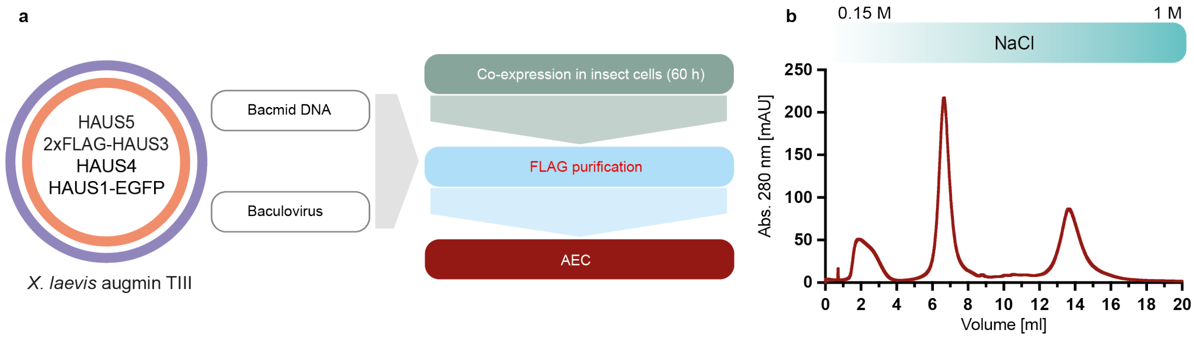

Supplementary Fig. 3: Recombinant expression and biochemical purification of the augmin tetramer TIII: a Augmin TIII tetramer purification scheme. MultiBac construct of the genes encoding HAUS proteins of the *X. laevis* augmin TIII tetramer used for baculovirus production (grey) and protein expression in insect cells (green). Complex was purified via FLAG purification (light blue) and anion exchange chromatography (AEC, red). **b** AEC chromatogram of Capto^{TM} HiRes Q 5/50 run. Complexes were eluted via a gradient from 150 mM NaCl to 1 M NaCl (turquoise gradient). Source data are provided as a Source Data file.

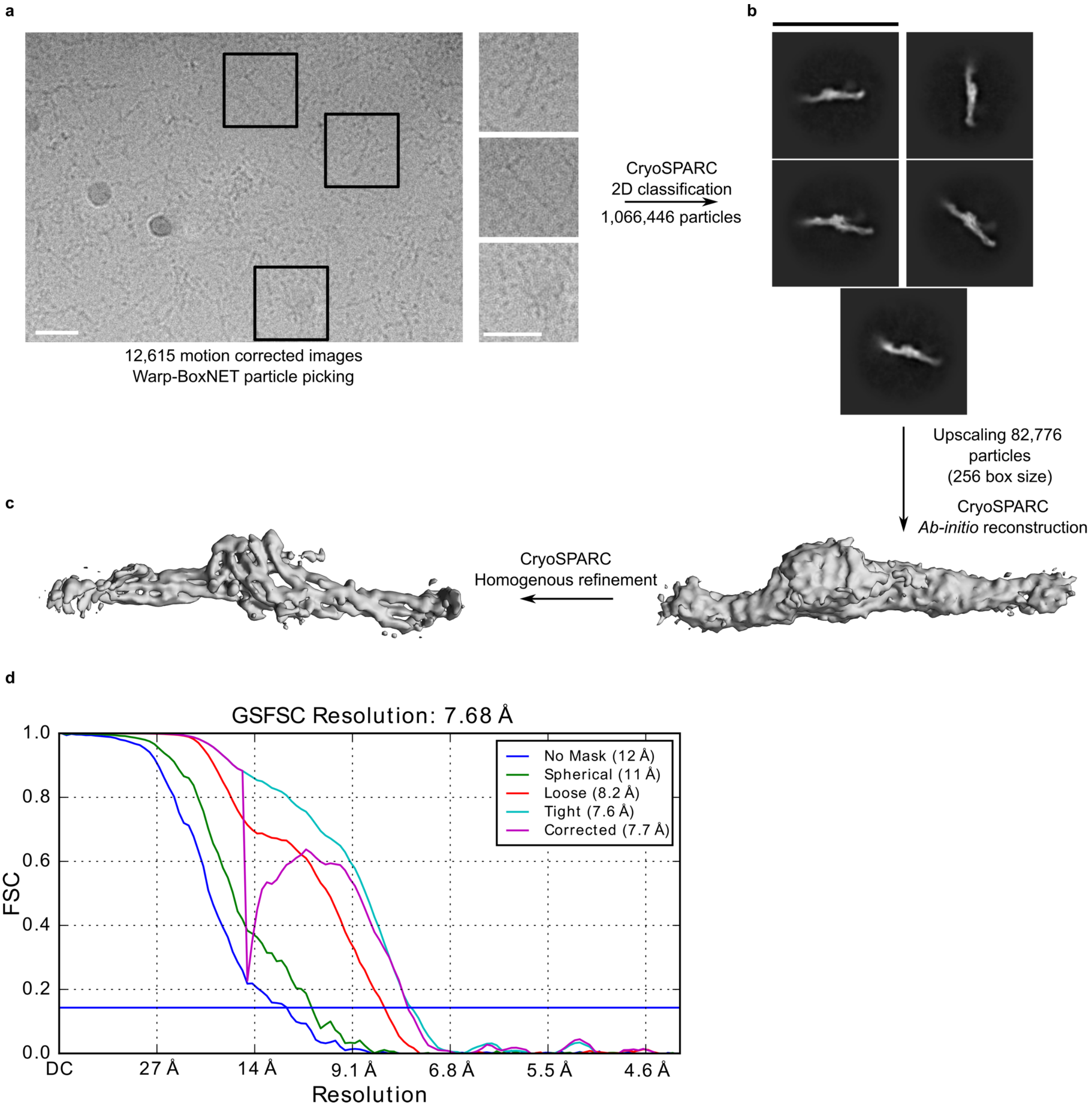

Supplementary Fig. 4: Cryo-EM data processing scheme. a Representative micrograph with 3 representative particles. Number of micrographs and picked particles for 2D classification are given. Scale bars: 30 nm. **b** 2D class averages containing 82,776 particles used for final 3D reconstructions. Scale bar: 54.8 nm. **c** Right: *ab-initio* reconstruction of the augmin TIII tetramer. Left: reconstruction after homogeneous refinement of the augmin TIII tetramer. **d** Gold Standard Fourier Shell Correlation plot for the homogeneous refinement from cryoSPARC. Source data are provided as a Source Data file.

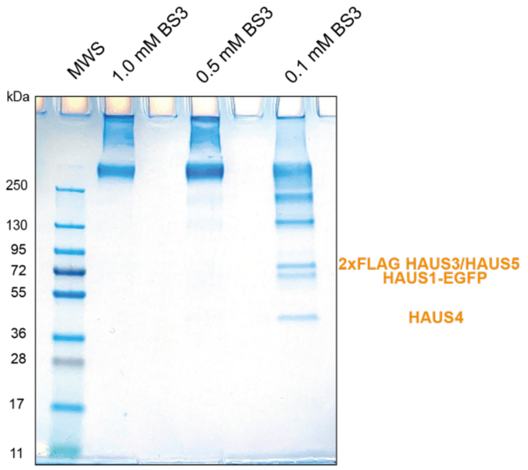

Supplementary Fig. 5: BS3 crosslinking of augmin tetramer TIII: Coomassie-stained SDS-PAGE of augmin TIII tetramer samples after crosslinking with varying BS3 concentrations. 0.1 mM BS3 was used for the final crosslinking experiment subjected to mass spectrometry (see methods, n=1 experiment). The BS3 crosslinking optimisation experiment with varying BS3 concentrations was repeated once with similar results (n=2 experiments). Source data are provided as a Source Data file.

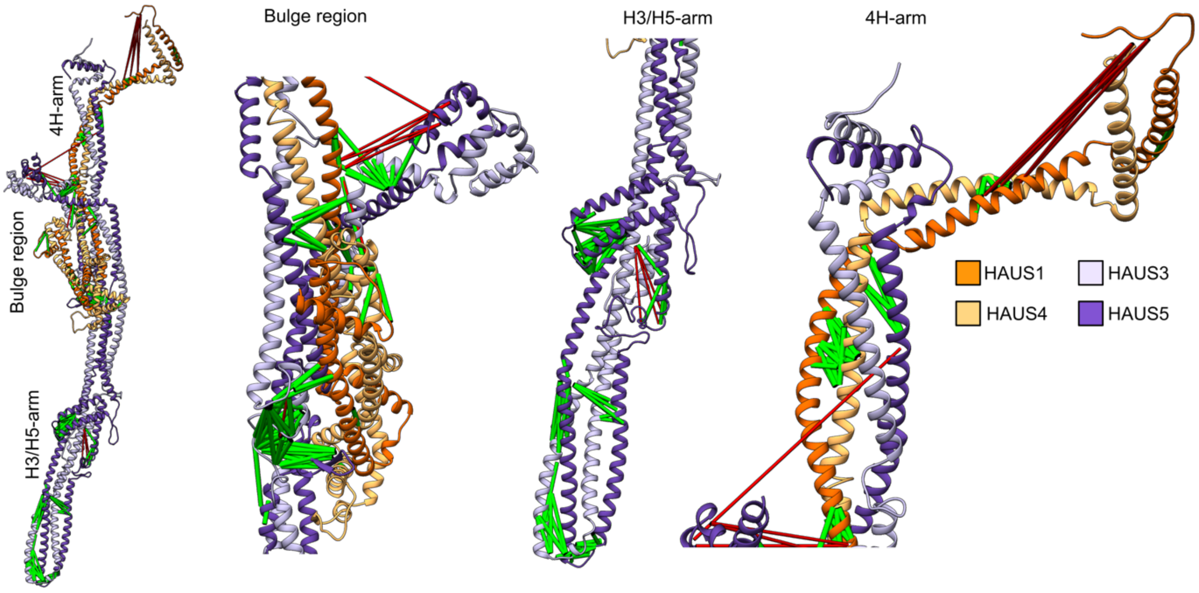

Supplementary Fig. 6: Details for validation of the predicted TIII model by crosslinking mass spectrometry. Left**:** Visualization of crosslinks used for model validation mapped back to the TIII model. Colouring scheme is given. Satisfied (green) and violated (red) crosslinks according to a distance threshold of 30 Å. Right: Zoomed views on different TIII regions.

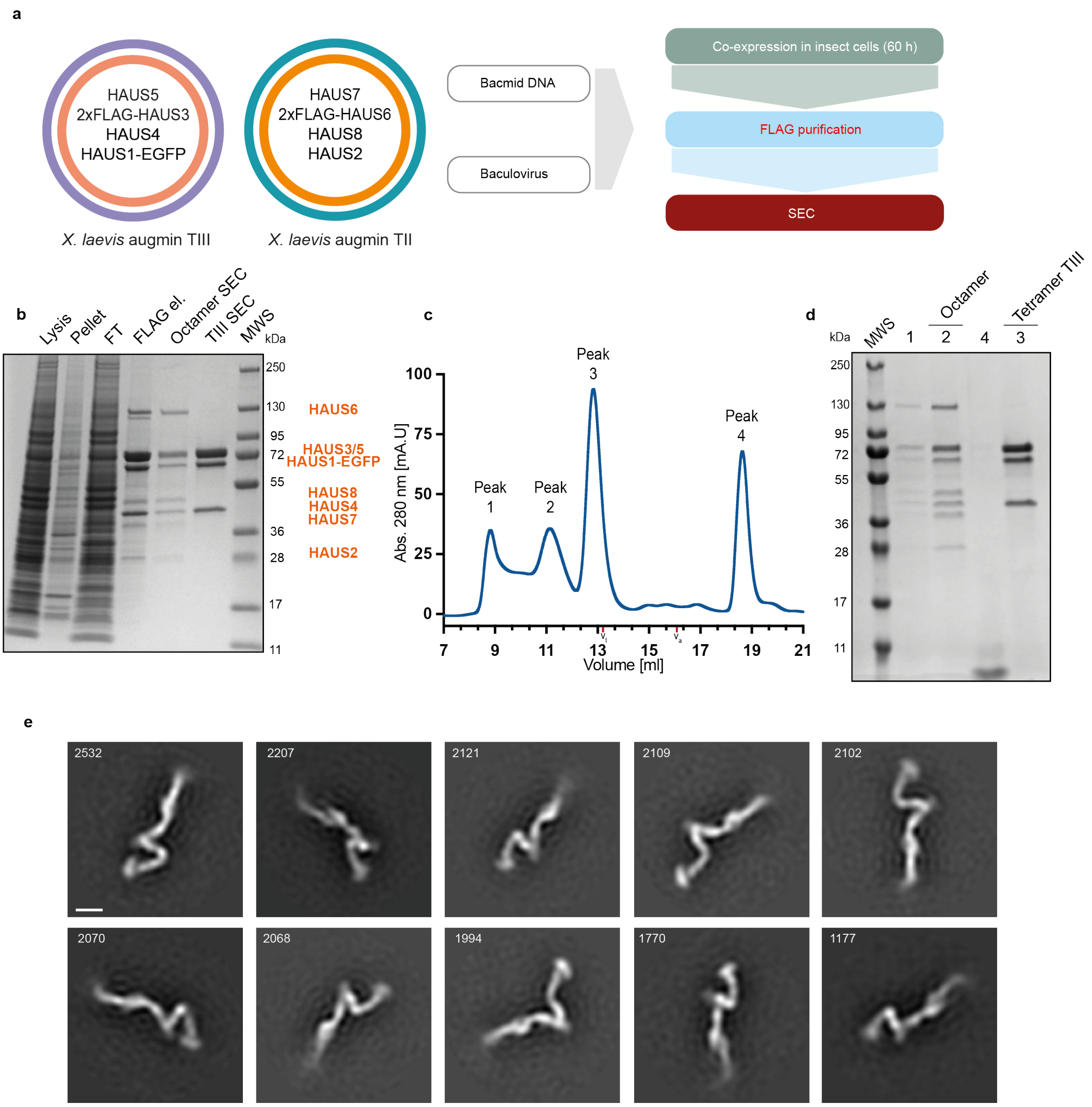

Supplementary Fig. 7: Recombinant expression, biochemical purification and negative stain EM analysis of the octameric augmin holocomplex. a Augmin octamer purification scheme. MultiBac constructs with the genes encoding HAUS proteins of the *X. laevis* augmin TIII and TII tetramers were used for baculovirus production (grey) and co-expression in insect cells (green). Complexes were purified via FLAG purification (light blue) and size exclusion chromatography (SEC, red). **b** SDS-PAGE analysis of the augmin octamer purification, MWS: molecular weight standard; Lysis: cell lysate; Pellet: cell pellet; FT: flow-through after incubation with FLAG beads: FLAG el.: FLAG elution; Octamer SEC: octamer peak after SEC (peak 2); TIII peak: TIII peak after SEC (peak 3). Purified proteins are indicated (orange). Augmin octamer purifications and SDS-PAGE analysis was repeated at least twice with similar results (n=3 experiments). **c** SEC chromatogram of Superose 6 increase (10/300) column run of augmin octamer FLAG elutions. Thyroglobulin 669 kDa (13.2 ml, v_t) and aldolase 158 kDa

(16.3 ml, v_a) were used in independent runs as size markers. **d** SDS-PAGE analysis of the four peak fractions from the SEC run (**c**). SEC on augmin octamer sample and SDS-PAGE analysis was repeated at least twice with similar results (n=3 experiments). **e** Representative negative stain EM 2D class averages of the augmin octamer SEC peak fraction. Scale bar:10 nm. Particle numbers contributing to the 2D classes are given. Source data are provided as a Source Data file.

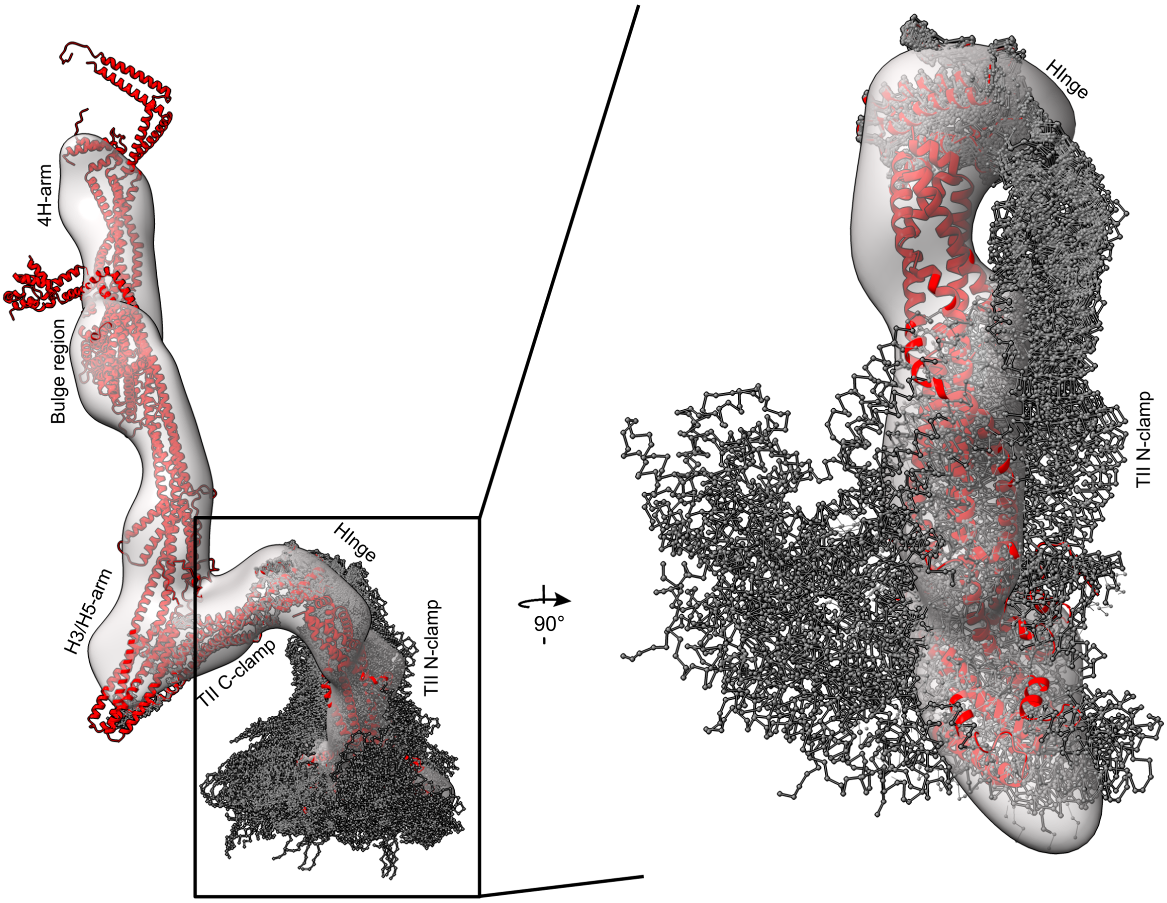

Supplementary Fig. 8: The predicted relative arrangement of TII N- and C-clamps is not compatible with the augmin holocomplex structure determined by negative stain EM. The ensemble of predicted models for the TII tetramer (grey; chain-trace representation) was superposed to the atomic model of augmin in the open conformation (red; ribbon representation) according to the TII C-clamp. The negative stain EM density of augmin in the open conformation was superposed (transparent grey). Right panel: Zoomed and rotated view of the region indicated in the left panel, with only the TII N-clamp shown.

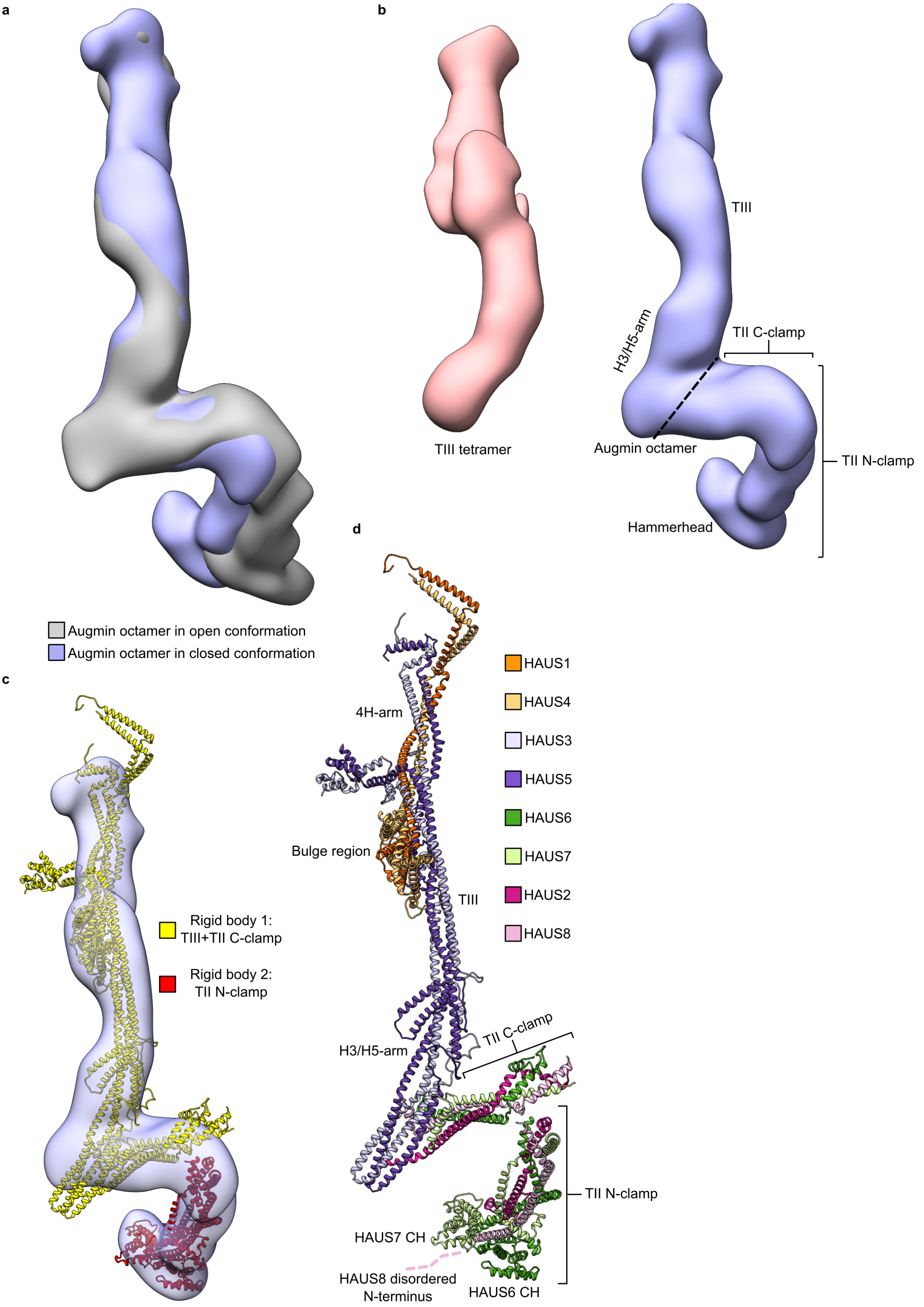

Supplementary Fig. 9: The augmin holocomplex in closed conformation. a Negative stain EM 3D reconstruction of the augmin holocomplex in two main conformations superposed according to TIII (open conformation: grey; closed conformation: purple). **b** Negative stain EM

3D reconstructions of the isolated TIII tetramer (red) and the augmin holocomplex in closed conformation (purple). Structural features are annotated and the boundary between TII and TIII in the augmin holocomplex is indicated (dashed line). **c** Two model segments were fitted into the negative stain EM 3D reconstruction of the augmin holocomplex in closed conformation as rigid bodies: TIII + TII C-clamp (yellow); TII N-clamp (red). **d** Subunit architecture of the complete augmin holocomplex in closed conformation. Structural features are indicated. Colour scheme is given.

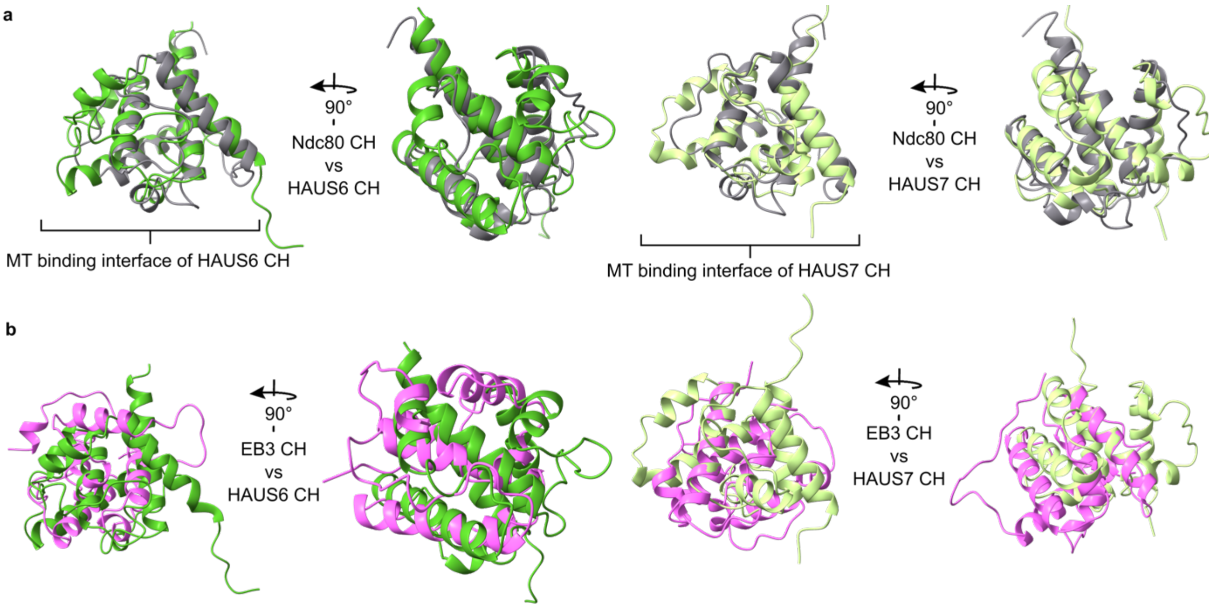

Supplementary Fig. 10: Structural similarity between the N-terminal globular domains of HAUS6/7 and microtubule-binding Calponin Homology (CH) domains. a Superposition of Ndc80-Calponin homology (CH) domain (grey; PDB 3IZ0) on the N-termini of HAUS6 (dark green; RMSD of 5.4 Å) and HAUS7 (bright green; RMSD of 4.6 Å). The MT binding interface is indicated. **b** Superposition of EB3-Calponin homology (CH) domain (purple; PDB 3JAR) on the N-termini of HAUS6 (dark green; RMSD of 16.9 Å) and HAUS7 (bright green; RMSD of 13.3 Å).

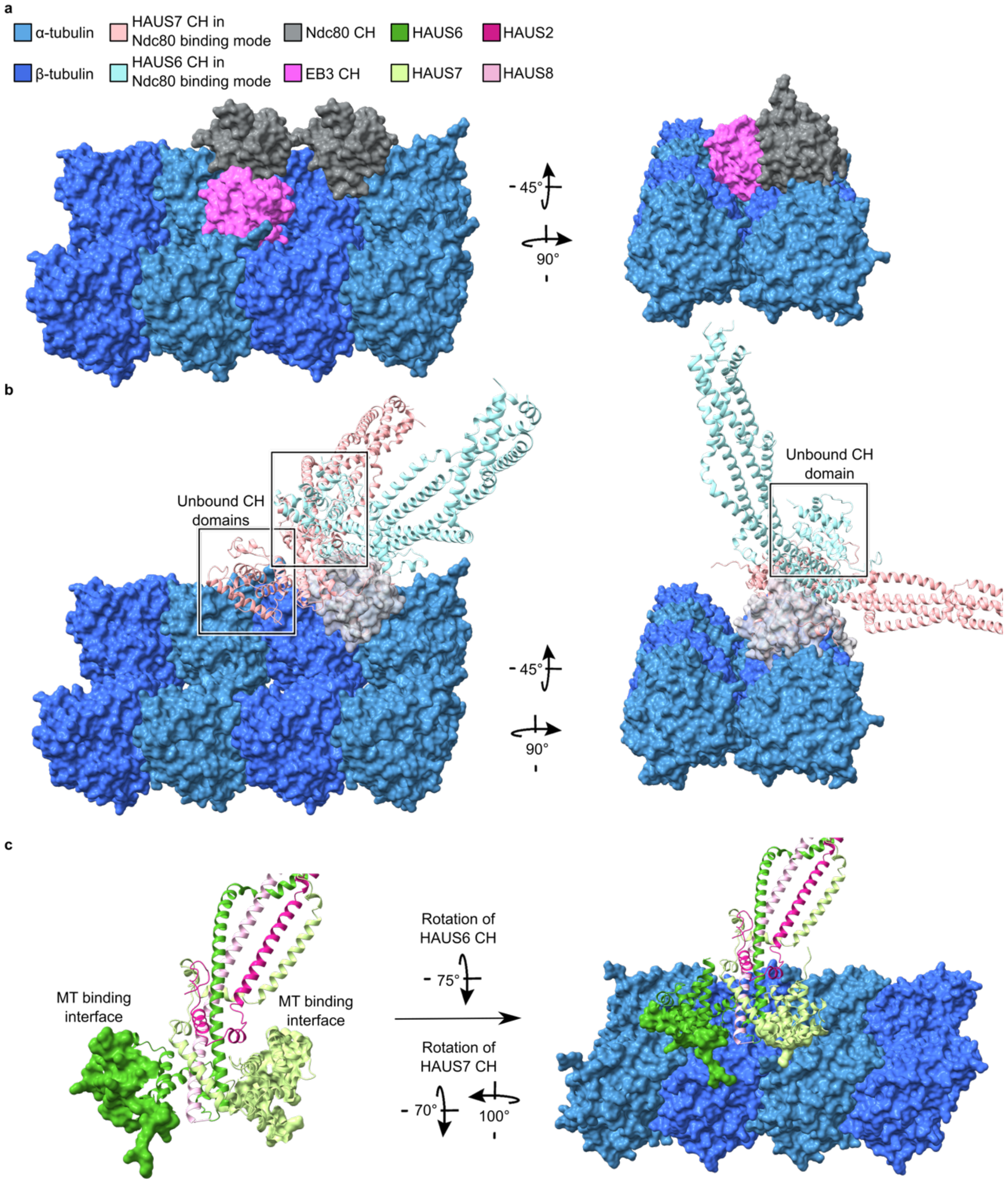

Supplementary Fig. 11: Structural details of CH domain binding to microtubules. a Structure of EB3 (purple) and Ndc80 CH domains (grey; PDB 3IZ0) bound to the microtubule lattice (PDB 6EW0). Colour scheme is given for all panels. **b** N-clamp orientation after superposition of the HAUS6 (light blue) or HAUS7 CH domain (salmon) to the Ndc80 CH domain on the microtubule lattice. Unbound CH domains are indicated. **c** TII N-clamp with the microtubule-binding interfaces of the HAUS6 and HAUS7 CH domains shown in molecular surface representation. Rotations of both CH domains around their centroids required for superposition to the Ndc80 CH domains on the microtubule lattice are indicated.

Supplementary Table 1: Predicted augmin TIII models ordered according to pLDDT score.

Model	pLDDT
Augmin TIII tetramer 1	0.625
Augmin TIII tetramer 2	0.617
Augmin TIII tetramer 3	0.611
Augmin TIII tetramer 4	0.611
Augmin TIII tetramer 5	0.610
Augmin TIII tetramer 6	0.608
Augmin TIII tetramer 7	0.608
Augmin TIII tetramer 8	0.607
Augmin TIII tetramer 9	0.607
Augmin TIII tetramer 10	0.604
Augmin TIII tetramer 11	0.603
Augmin TIII tetramer 12	0.602
Augmin TIII tetramer 13	0.602
Augmin TIII tetramer 14	0.600
Augmin TIII tetramer 15	0.597
Augmin TIII tetramer 16	0.597
Augmin TIII tetramer 17	0.596
Augmin TIII tetramer 18	0.594
Augmin TIII tetramer 19	0.593
Augmin TIII tetramer 20	0.593
Augmin TIII tetramer 21	0.593
Augmin TIII tetramer 22	0.591
Augmin TIII tetramer 23	0.591
Augmin TIII tetramer 24	0.59
Augmin TIII tetramer 25	0.588

Supplementary Table 2: Predicted augmin TII models ordered according to pLDDT score.

Model	pLDDT
Augmin TII tetramer 1	0.569
Augmin TII tetramer 2	0.564
Augmin TII tetramer 3	0.561
Augmin TII tetramer 4	0.559
Augmin TII tetramer 5	0.559
Augmin TII tetramer 6	0.558
Augmin TII tetramer 7	0.556
Augmin TII tetramer 8	0.556
Augmin TII tetramer 9	0.555
Augmin TII tetramer 10	0.554
Augmin TII tetramer 11	0.550
Augmin TII tetramer 12	0.549
Augmin TII tetramer 13	0.548
Augmin TII tetramer 14	0.542
Augmin TII tetramer 15	0.541
Augmin TII tetramer 16	0.541
Augmin TII tetramer 17	0.540
Augmin TII tetramer 18	0.536
Augmin TII tetramer 19	0.535
Augmin TII tetramer 20	0.533
Augmin TII tetramer 21	0.533
Augmin TII tetramer 22	0.530
Augmin TII tetramer 23	0.530
Augmin TII tetramer 24	0.530
Augmin TII tetramer 25	0.528

Supplementary Table 3: EM data collection and refinement statistics

Data collection and processing			
Sample	Augmin TIII tetramer	Augmin holocomplex	
EMDB ID	15631	15632	15633
PDB ID	8AT2	8AT3	8AT4
Microscope	Titan Krios G1	Talos L120C	Talos L120C
Detector	Gatan K3 camera	4K Ceta CMOS	4K Ceta CMOS
Magnification	81,000	57,000	57,000
Voltage (kV)	300	120	120
Electron exposure ($e^-/Å^2$)	68.9	101.8	101.8
Defocus range (μm)	-1 to -3	-2 to -3	-2 to -3
Pixel size (Å/px)	1.07	2.552	2.552
Symmetry imposed	C1	C1	C1
Number of movies/micrographs	12,615	583	583
Initial particle images	1,060,446	80,837	80,837
Final particle images	82,776	11,969	10,658
Map resolution (Å)	7.7	33	33
FSC threshold	0.143	0.143	0.143
Map-sharpening B factor ($Å^2$)	-797.4	0	0
Model building and refinement			
Model composition			
Protein atoms	15,357	24,599	24,599
Nucleic acid atoms	0	0	0
Ligands	0	0	0
RMSDs from ideal			
Bond lengths (Å)	0.035	0.024	0.024
Bond angles (°)	3.432	2.444	2.445
Validation			
Clashscore	1.10	31.50	29.65
Rotamer outliers (%)	2.48	5.78	6.10
Ramachandran plot			
Favoured (%)	95.78	94.34	94.48
Allowed (%)	2.53	2.38	2.31
Outlier (%)	1.69	3.28	3.21

Supplementary Table 4: Cross-correlation of augmin TIII models fitted into the TIII cryo-EM density

Model	Correlation
Augmin TIII tetramer 1	0.7316
Augmin TIII tetramer 2	0.7334
Augmin TIII tetramer 3	0.7394
Augmin TIII tetramer 4	0.7235
Augmin TIII tetramer 5	0.7332
Augmin TIII tetramer 6	0.7389
Augmin TIII tetramer 7	0.7287
Augmin TIII tetramer 8	0.7397
Augmin TIII tetramer 9	0.7322
Augmin TIII tetramer 10	0.7503
Augmin TIII tetramer 11	0.7176
Augmin TIII tetramer 12	0.7454
Augmin TIII tetramer 13	0.7285
Augmin TIII tetramer 14	0.6482
Augmin TIII tetramer 15	0.7078
Augmin TIII tetramer 16	0.7084
Augmin TIII tetramer 17	0.7124
Augmin TIII tetramer 18	0.7453
Augmin TIII tetramer 19	0.7194
Augmin TIII tetramer 20	0.7220
Augmin TIII tetramer 21	0.7167
Augmin TIII tetramer 22	0.7303
Augmin TIII tetramer 23	0.7323
Augmin TIII tetramer 24	0.7187
Augmin TIII tetramer 25	0.7130

Supplementary Table 5: RMSD towards the highest scored model.

Model	RMSD
Augmin TIII tetramer 2	8.259
Augmin TIII tetramer 3	6.262
Augmin TIII tetramer 4	19.271
Augmin TIII tetramer 5	6.311
Augmin TIII tetramer 6	4.579
Augmin TIII tetramer 7	21.706
Augmin TIII tetramer 8	16.455
Augmin TIII tetramer 9	6.782
Augmin TIII tetramer 10	19.175
Augmin TIII tetramer 11	9.264
Augmin TIII tetramer 12	17.070
Augmin TIII tetramer 13	7.720
Augmin TIII tetramer 14	31.214
Augmin TIII tetramer 15	7.246
Augmin TIII tetramer 16	12.756
Augmin TIII tetramer 17	8.411
Augmin TIII tetramer 18	19.900
Augmin TIII tetramer 19	11.674
Augmin TIII tetramer 20	22.879
Augmin TIII tetramer 21	20.538
Augmin TIII tetramer 22	19.234
Augmin TIII tetramer 23	21.358
Augmin TIII tetramer 24	7.611
Augmin TIII tetramer 25	19.629

Supplementary Table 6: Predicted augmin TII+H3/H5 arm models ordered according to pLDDT score.

Model	pLDDT
Augmin TII +H3/H5 arm 1	0.512
Augmin TII +H3/H5 arm 2	0.511
Augmin TII +H3/H5 arm 3	0.509
Augmin TII +H3/H5 arm 4	0.507
Augmin TII +H3/H5 arm 5	0.506
Augmin TII +H3/H5 arm 6	0.506
Augmin TII +H3/H5 arm 7	0.506
Augmin TII +H3/H5 arm 8	0.503
Augmin TII +H3/H5 arm 9	0.503
Augmin TII +H3/H5 arm 10	0.496
Augmin TII +H3/H5 arm 11	0.477
Augmin TII +H3/H5 arm 12	0.477
Augmin TII +H3/H5 arm 13	0.474
Augmin TII +H3/H5 arm 14	0.473
Augmin TII +H3/H5 arm 15	0.469
Augmin TII +H3/H5 arm 16	0.469
Augmin TII +H3/H5 arm 17	0.468
Augmin TII +H3/H5 arm 18	0.467
Augmin TII +H3/H5 arm 19	0.467
Augmin TII +H3/H5 arm 20	0.465
Augmin TII +H3/H5 arm 21	0.464
Augmin TII +H3/H5 arm 22	0.463
Augmin TII +H3/H5 arm 23	0.462
Augmin TII +H3/H5 arm 24	0.46
Augmin TII +H3/H5 arm 25	0.458

Supplementary Table 7: Sequence identity of CH domains amongst different MT-binding proteins

Protein Name	Sequence identity (%)					
	HAUS7	HAUS6	Ndc80/Hec1	Nuf2	EB1	EB3
HAUS7	100	11.43	22.43	10.74	17.78	16.3
HAUS6	11.43	100	13.11	14.6	14.81	14.81
Ndc80/Hec1	22.43	13.11	100	15.65	13.39	12.28
Nuf2	10.74	14.6	15.65	100	15.00	13.33
EB1	17.78	14.81	13.39	15.00	100	80.41
EB3	16.3	14.81	12.28	13.33	80.41	100

Supplementary Table 8: Primer list.

Name	Sequence (5' → 3')	Source	Identifier
MultiBac_vector_fwd	TCTAGAGCCTGCAGTCTCG	Würtz et al., 2021	N/A
MultiBac_vector_rev	CAGTTTTGTAATAAAAAAACCTATAAATAT	Würtz et al., 2021	N/A
Combination_vector_fwd	TTCGCGACCTACTCCGGA	Würtz et al., 2021	N/A
Combination_vector_rev	CAGATAACTTCGTATAATGTATGCT	Würtz et al., 2021	N/A
Combination_insert_fwd	ATACGAAGTTATCTGTTCGCGACCTACTCCGGA	Würtz et al., 2021	N/A
Combination_insert_rev	GGAGTAGGTCGCGAAGATCCAGACATGATAAGATACA TTG	Würtz et al., 2021	N/A
Pet26b-EGFP fwd	GGATCCGCTGGCTCCGCT	This study	N/A
Pet26b-EGFP rev	TATTTCTAGAGGGGAATTGTTATCCGCTCACAATTC	This study	N/A
HAUS1-EGFP_fwd	taataaaaaaacctataaatatGGACGAGAAGAGCACTAAG	This study	N/A
HAUS1-EGFP_rev	tcgagactgcaggctctagaTTAGTGATGGTGATGGTG	This study	N/A
HAUS8_fwd	taataaaaaaacctataaatatGTCGGAAGCTGGAGTTGC	This study	N/A
HAUS8_rev	gactgcaggctctagaATCAAGGTAGGGATCCATCAAAATAC	This study	N/A

Supplementary Table 9: Pet26b-EGFP DNA sequence

5`-

GTCTGCTCCCGGCATCCGCTTACAGACAAGCTGTGACCGTCTCCGGGAGCTGCATGTGTCAGAGGTTTTCACCGTCATCACCGAAACGCGCGAGGCAG
CTGCGGTAAAGCTCATCAGCGTGGTCGTGAAGCGATTCACAGATGTCTGCCTGTTCATCCGCGTCCAGCTCGTTGAGTTTCTCCAGAAGCGTTAATGTCT
GGCTTCTGATAAAGCGGGCCATGTTAAGGGCGGTTTTTTCCTGTTTGGTCACTGATGCCTCCGTGTAAGGGGGATTTCTGTTCATGGGGGTAATGATACC
GATGAAACGAGAGAGGATGCTCACGATACGGGTTACTGATGATGAACATGCCCGGTTACTGGAACGTTGTGAGGGTAAACAACTGGCGGTATGGATGCG
GCGGGACCAGAGAAAAATCACTCAGGGTCAATGCCAGCGCTTCGTTAATACAGATGTAGGTGTTCCACAGGGTAGCCAGCAGCATCCTGCGATGCAGAT
CCGGAACATAATGGTGCAGGGCGCTGACTTCCGCGTTTCCAGACTTTACGAAACACGGAAACCGAAGACCATTCATGTTGTTGCTCAGGTCGCAGACGTT
TTGCAGCAGCAGTCGCTTCACGTTCGCTCGCGTATCGGTGATTCATTCTGCTAACCAGTAAGGCAACCCCGCCAGCCTAGCCGGGTCCTCAACGACAGG
AGCACGATCATGCGCACCCGTGGGGCCGCCATGCCGGCGATAATGGCCTGCTTCTCGCCGAAACGTTTGGTGGCGGGACCAGTGACGAAGGCTTGAG
CGAGGGCGTGCAAGATTCCGAATACCGCAAGCGACAGGCCGATCATCGTCGCGCTCCAGCGAAAGCGGTCCTCGCCGAAAATGACCCAGAGCGCTGCC
GGCACCTGTCCTACGAGTTGCATGATAAAGAAGACAGTCATAAGTGCGGCGACGATAGTCATGCCCCGCGCCCACCGGAAGGAGCTGACTGGGTTGAA
GGCTCTCAAGGGCATCGGTCGAGATCCCGGTGCCTAATGAGTGAGCTAACTTACATTAATTGCGTTGCGCTCACTGCCCGCTTTCCAGTCGGGAAACCT
GTCGTGCCAGCTGCATTAATGAATCGGCCAACGCGCGGGGAGAGGCGGTTTGCGTATTGGGCGCCAGGGTGGTTTTTCTTTTCACCAGTGAGACGGGC
AACAGCTGATTGCCCTTCACCGCCTGGCCCTGAGAGAGTTGCAGCAAGCGGTCCACGCTGGTTTGCCCCAGCAGGCGAAAATCCTGTTTGATGGTGGTT
AACGGCGGGATATAACATGAGCTGTCTTCGGTATCGTCGTATCCCACTACCGAGATATCCGCACCAACGCGCAGCCCGGACTCGGTAATGGCGCGCATT
GCGCCCAGCGCCATCTGATCGTTGGCAACCAGCATCGCAGTGGGAACGATGCCCTCATTCAGCATTTGCATGGTTTGTTGAAAACCGGACATGGCACTC
CAGTCGCCTTCCCGTTCCGCTATCGGCTGAATTTGATTGCGAGTGAGATATTTATGCCAGCCAGCCAGACGCAGACGCGCCGAGACAGAACTTAATGGG
CCCGCTAACAGCGCGATTTGCTGGTGACCCAATGCGACCAGATGCTCCACGCCCAGTCGCGTACCGTCTTCATGGGAGAAAATAATACTGTTGATGGGT
GTCTGGTCAGAGACATCAAGAAATAACGCCGGAACATTAGTGCAGGCAGCTTCCACAGCAATGGCATCCTGGTCATCCAGCGGATAGTTAATGATCAGCC
CACTGACGCGTTGCGCGAGAAGATTGTGCACCGCCGCTTTACAGGCTTCGACGCCGCTTCGTTCTACCATCGACACCACCACGCTGGCACCCAGTTGAT
CGGCGCGAGATTTAATCGCCGCGACAATTTGCGACGGCGCGTGCAGGGCCAGACTGGAGGTGGCAACGCCAATCAGCAACGACTGTTTGCCCGCCAGT
TGTTGTGCCACGCGGTTGGGAATGTAATTCAGCTCCGCCATCGCCGCTTCCACTTTTTCCCGCGTTTTCGCAGAAACGTGGCTGGCCTGGTTCACCACG
CGGGAAACGGTCTGATAAGAGACACCGGCATACTCTGCGACATCGTATAACGTTACTGGTTTCACATTCACCACCCTGAATTGACTCTCTTCCGGGCGCT
ATCATGCCATACCGCGAAAGGTTTTGCGCCATTCGATGGTGTCCGGGATCTCGACGCTCTCCCTTATGCGACTCCTGCATTAGGAAGCAGCCCAGTAGTA
GGTTGAGGCCGTTGAGCACCGCCGCCGCAAGGAATGGTGCATGCAAGGAGATGGCGCCCAACAGTCCCCCGGCCACGGGGCCTGCCACCATACCCAC
GCCGAAACAAGCGCTCATGAGCCCGAAGTGGCGAGCCCGATCTTCCCCATCGGTGATGTCGGCGATATAGGCGCCAGCAACCGCACCTGTGGCGCCG
GTGATGCCGGCCACGATGCGTCCGGCGTAGAGGATCGAGATCTCGATCCCGCGAAATTAATACGACTCACTATAGGGGAATTGTGAGCGGATAACAATT
CCCCTCTAGAAATAATTTTGTTTAACTTTAAGAAGGAGATATACATATGATGCAGCCCCCTGCAGTTGCACAGGCATCCGCTTCACCCCCACCAGCTGCTT
CTTCTGATTCTGGATCTTCGACATCGGATTACAAACCAGATCCTAAGAAAACCAAGCCAGGAACTCAAGCATCCAAAGCGAAGACCCAGTCGGTCTCCTC
TGAAGGTAATACTAGTTTGAATCCATCCAATACTAGTTTGACTCCATCCAAAGCTAACACCAGCCTGAGCAAAGCCAAACCGGCTAAACAGACCCTGCCTG
GAAAGAAAGCTCCAAGCAAGCCAAATGCAAAGGACGAGGAGGACAAGTCTGGGCCAATCTACATAATTGTTCCAAATGGGAAGGAGCAGAGAGTGAAGG
ACGAGAAGGCACTGAAGGTCCTCAAATGGAATTTCACTACCCCGCGTGATGAATATATTGAACAACTCAAGACACAGATGTCCCCATGTATTGCCAGATG
GCTGCAGGATGAGCTGTTCCATGCCGACTTCCAGCGCCAAATCAAAGGCTTGGCCGTAATGACTGAGCATTTGGAAAGTGAGAAAGAGGGGGTTATCAG
CTGCTTGGACCTCGTTTTGAAGTGGTTCACCCTTCGATTCTTCGATACGAACACAAGTGTCCTGATGAAGTGTCTGGAGTATCTCAAGCTGCTCTTCATCA
TGCTCAGCCAGGAGGAGTACCATCTGACAGAGATGGAGGGCACCTCCTTTCTTCCTTACCTAATGCTGAAGGTGGGAGAACCAAAGGACATAGTGCGGA
AAGATGTACGTGCCATCCTAACTAAAATGTGCCAGGTGTATCCTGCCAGCAAGATGTTTAACTTTGTGATGGAAGGGACCAAATCAAAGAACTCCAAGCA
GCGAGCTGAGTGCCTGGAGGAGCTGGGTTGCTTGGTGGAGTCTTACGGTATGAATGTGTGCCAGCCCACACCTGCCAAAGCTCTGAAGGAGATCGCTAT
ACATATAGGGGACAGAGACACGACTGTGCGTAACGCTGCCCTCAACACTATAGTGACAGTGTACAACGTTCATGGAGAACAGGTCTTCAAACTCATTGGC
AATCTTTCAGAGAAGGACATGAGCATGCTAGAGGAGAGGATCAAACGTGCAGGCAAGAAGCAAGCTGCGGCGGCTCCAGCTAAGCAAGTGGAGGAGAA
ACCTCAGCGTGTGCAGAGTGCCAATGCCAGCATCCTACGCAAAGCACCCCCGGAAGACATGTCCTCCAAACTGAATCAAGCCCGAAACATGGGAGGCCA
CACTGAGCCGTCTCACTCTGTCCCGCGGGAGTTCCAGTTGGACCTGGATGAGATTGAGAATGACAATGGCACAGTCCGATGTGAGATGCCGGCACTTGT
ACAGCACAAACTGGACGAGATATTTGAGCCAGTCCTAATCCCGGAACCAAAGATCCGTGCCGTGTCTCCGCACTTTGATGACATGCACAGTAACACGGCT
TCCACCATCAACTTTGTAATCTCCCAGGTGGCCAGCGTAGACATCAATGCCAGCATTCAAGCCCTTGCACAGATTGATGAGGTGCTGAGGCAGGAAGATA
AGGCTGAAGCCATGTCTGGCCACATTGACCAGTTCCTCATTGCCACCTTCATGCAACTGCGTTTGGCCTACAACACTCACATGGCAGATGAGCGGCTGGA
TAAGGACGATATTGTGCGCTTGTATAGTTGCATCATCGGGAACATGATCTCGTTATTCCAGATGGAAAGCCTGGCCAGGGAAGCTTCTACTGGAGTGCTG
AAGGACTTAATGCACGGCCTTATTAGCCTGATGCTGGATGCTCGAATAGAGGATCTCGAGGAGGGGCAGCAGGTGGTGCGCTCTGTTAATTTGTTGGTG
GTGAAGGTGCTGGAGAAGTCGGACCAGACCAATATCATAAGTGCTCTGCTTATGCTGCTCCAGGATAGCCTTCTGGCTACAGCGAGTTCCCCCAATTTCT
CCGAGCTGGTTATGAAGTGTCTTTGGCGAATGATTCGTCTCCTGCCAGAGGCCATAAACAACCTCAATCTGGATAGGATTCTGCTGGACATCCATAACTTC
ATGAGGGTCCTACCCAAGGAAAAGCTAAAGCAGCACAAGAGCGAGATGCCTATGAGGACTCTGAAAACTCTCCTACACACACTTTGCAAGCTAAAAGGGC
CCAAAATCATGGACCACCTGAGTATGATTGAGAACAAACATGAGTCTGAGTTGGAGGCCCATCTCCTCAGGGTGATGAAGCACTCCATAGACCGAACTGG
TTCAAAGGGCGACAAGGAGACTGAGAAGGGAGCATCTTGCATTGAAGACAAGGTGGGAAAAGCAAATGTGAGTGACTTTCTTGCTGAAATGTTTAAGAAG
ATTGGCTCTAAAGAGAACACTAAAGAGGGCCTGGCAGAACTCTACGAGTATAAAAAGAAATACTCTGATGCAGACATCAAGCCATTCCTCAAGAACTCCTC
GCAGTTCTTCCAGAGCTATGTAGAGCGGGGCCTCCGGCTCATAGAGATGGAAAGGGAGGGCAAAGCCAGAATAGCCCCAAACACAGGAATGTCAACCC
ATGTGACAGAGATGACCCCTCTCCCTACAGTGACCAATACAGCAGCTCCTGTTTCAAACACAAATGGGGAGGAAGTCGGGCCCTCCGTTTACCTGGAGC
GGCTGAAAATTCTGCGCCAGAGATGTGGGCTTGACAATGCCAAGCAGGACGAGAGGCCCCCATTGACCTCGTTACTTTCAAAGTCTTCTGCCCCCGCCG
TCGTGTCCTCCACCGACATGCTACACAGCAAACTCTCCCAACTGCGAGAGTCACGGGAACAATTTCAGCATGTCGAACTCGACTCCAACCAGACCTACCC
TAGCACAACCACCTCATCCTCCGCCTCTTCAACAAACATCGACGACTTGAAAAAGAGACTGGAAAGGATCAAAAGTAGCCGGAAAGGATCCGCTGGCTCC
GCTGCTGGTTCTGGCGAATTCATGGTGAGCAAGGGCGAGGAGCTGTTCACCGGGGTGGTGCCCATCCTGGTCGAGCTGGACGGCGACGTAAACGGCC
ACAAGTTCAGCGTGTCCGGCGAGGGCGAGGGCGATGCCACCTACGGCAAGCTGACCCTGAAGTTCATCTGCACCACCGGCAAGCTGCCCGTGCCCTG
GCCCACCCTCGTGACCACCCTGACCTACGGCGTGCAGTGCTTCAGCCGCTACCCCGACCACATGAAGCAGCACGACTTCTTCAAGTCCGCCATGCCCG
AAGGCTACGTCCAGGAGCGCACCATCTTCTTCAAGGACGACGGCAACTACAAGACCCGCGCCGAGGTGAAGTTCGAGGGCGACACCCTGGTGAACCGC
ATCGAGCTGAAGGGCATCGACTTCAAGGAGGACGGCAACATCCTGGGGCACAAGCTGGAGTACAACTACAACAGCCACAACGTCTATATCATGGCCGAC

AAGCAGAAGAACGGCATCAAGGTGAACTTCAAGATCCGCCACAACATCGAGGACGGCAGCGTGCAGCTCGCCGACCACTACCAGCAGAACACCCCCAT
CGGCGACGGCCCCGTGCTGCTGCCCGACAACCACTACCTGAGCACCCAGTCCGCCCTGAGCAAAGACCCCAACGAGAAGCGCGATCACATGGTCCTGC
TGGAGTTCGTGACCGCCGCCGGGATCACTCTCGGCATGGACGAGCTGTACAAGCTGGAAGTTCTGTTCCAGGGGCCCCACCACCATCACCATCACCATC
ACTAATAGCTCGAGCACCACCACCACCACCACTGAGATCCGGCTGCTAACAAAGCCCGAAAGGAAGCTGAGTTGGCTGCTGCCACCGCTGAGCAATAAC
TAGCATAACCCCTTGGGGCCTCTAAACGGGTCTTGAGGGGTTTTTTGCTGAAAGGAGGAACTATATCCGGATTGGCGAATGGGACGCGCCCTGTAGCGG
CGCATTAAGCGCGGCGGGTGTGGTGGTTACGCGCAGCGTGACCGCTACACTTGCCAGCGCCCTAGCGCCCGCTCCTTTCGCTTTCTTCCCTTCCTTTCT
CGCCACGTTCGCCGGCTTTCCCCGTCAAGCTCTAAATCGGGGGCTCCCTTTAGGGTTCCGATTTAGTGCTTTACGGCACCTCGACCCCAAAAAACTTGAT
TAGGGTGATGGTTCACGTAGTGGGCCATCGCCCTGATAGACGGTTTTTCGCCCTTTGACGTTGGAGTCCACGTTCTTTAATAGTGGACTCTTGTTCCAAA
CTGGAACAACACTCAACCCTATCTCGGTCTATTCTTTTGATTTATAAGGGATTTTGCCGATTTCGGCCTATTGGTTAAAAAATGAGCTGATTTAACAAAAATT
TAACGCGAATTTTAACAAAATATTAACGTTTACAATTTCAGGTGGCACTTTTCGGGGAAATGTGCGCGGAACCCCTATTTGTTTATTTTTCTAAATACATTCA
AATATGTATCCGCTCATGAATTAATTCTTAGAAAAACTCATCGAGCATCAAATGAAACTGCAATTTATTCATATCAGGATTATCAATACCATATTTTTGAAAAA
GCCGTTTCTGTAATGAAGGAGAAAACTCACCGAGGCAGTTCCATAGGATGGCAAGATCCTGGTATCGGTCTGCGATTCCGACTCGTCCAACATCAATACA
ACCTATTAATTTCCCCTCGTCAAAAATAAGGTTATCAAGTGAGAAATCACCATGAGTGACGACTGAATCCGGTGAGAATGGCAAAAGTTTATGCATTTCTTT
CCAGACTTGTTCAACAGGCCAGCCATTACGCTCGTCATCAAAATCACTCGCATCAACCAAACCGTTATTCATTCGTGATTGCGCCTGAGCGAGACGAAATA
CGCGATCGCTGTTAAAAGGACAATTACAAACAGGAATCGAATGCAACCGGCGCAGGAACACTGCCAGCGCATCAACAATATTTTCACCTGAATCAGGATA
TTCTTCTAATACCTGGAATGCTGTTTTCCCGGGGATCGCAGTGGTGAGTAACCATGCATCATCAGGAGTACGGATAAAATGCTTGATGGTCGGAAGAGGC
ATAAATTCCGTCAGCCAGTTTAGTCTGACCATCTCATCTGTAACATCATTGGCAACGCTACCTTTGCCATGTTTCAGAAACAACTCTGGCGCATCGGGCTT
CCCATACAATCGATAGATTGTCGCACCTGATTGCCCGACATTATCGCGAGCCCATTTATACCCATATAAATCAGCATCCATGTTGGAATTTAATCGCGGCC
TAGAGCAAGACGTTTCCCGTTGAATATGGCTCATAACACCCCTTGTATTACTGTTTATGTAAGCAGACAGTTTTATTGTTCATGACCAAAATCCCTTAACGT
GAGTTTTCGTTCCACTGAGCGTCAGACCCCGTAGAAAAGATCAAAGGATCTTCTTGAGATCCTTTTTTTCTGCGCGTAATCTGCTGCTTGCAAACAAAAAA
ACCACCGCTACCAGCGGTGGTTTGTTTGCCGGATCAAGAGCTACCAACTCTTTTTCCGAAGGTAACTGGCTTCAGCAGAGCGCAGATACCAAATACTGTC
CTTCTAGTGTAGCCGTAGTTAGGCCACCACTTCAAGAACTCTGTAGCACCGCCTACATACCTCGCTCTGCTAATCCTGTTACCAGTGGCTGCTGCCAGTG
GCGATAAGTCGTGTCTTACCGGGTTGGACTCAAGACGATAGTTACCGGATAAGGCGCAGCGGTCGGGCTGAACGGGGGGTTCGTGCACACAGCCCAGC
TTGGAGCGAACGACCTACACCGAACTGAGATACCTACAGCGTGAGCTATGAGAAAGCGCCACGCTTCCCGAAGGGAGAAAGGCGGACAGGTATCCGGT
AAGCGGCAGGGTCGGAACAGGAGAGCGCACGAGGGAGCTTCCAGGGGGAAACGCCTGGTATCTTTATAGTCCTGTCGGGTTTCGCCACCTCTGACTTG
AGCGTCGATTTTTGTGATGCTCGTCAGGGGGGCGGAGCCTATGGAAAAACGCCAGCAACGCGGCCTTTTTACGGTTCCTGGCCTTTTGCTGGCCTTTTG
CTCACATGTTCTTTCCTGCGTTATCCCCTGATTCTGTGGATAACCGTATTACCGCCTTTGAGTGAGCTGATACCGCTCGCCGCAGCCGAACGACCGAGCG
CAGCGAGTCAGTGAGCGAGGAAGCGGAAGAGCGCCTGATGCGGTATTTTCTCCTTACGCATCTGTGCGGTATTTCACACCGCATATATGGTGCACTCTC
AGTACAATCTGCTCTGATGCCGCATAGTTAAGCCAGTATACACTCCGCTATCGCTACGTGACTGGGTCATGGCTGCGCCCCGACACCCGCCAACACCCG
CTGACGCGCCCTGACGGGCTT -3`
