# Supplementary material for: The augmin complex architecture reveals structural insights into microtubule branching
Source: Nat Commun. 2022 Sep 26;13:5635. doi: 10.1038/s41467-022-33228-6 (PMC9512787; doi:10.1038/s41467-022-33228-6)
Supplement: Supplementary file 3 — Description of Additional Supplementary Files [file 41467_2022_33228_MOESM3_ESM.pdf]

Description of Additional Supplementary Files

File Name: Supplementary Data 1
Description: List of unique potential crosslink sites from all individual replicates after manual validation and 5 % FDR cut-off.

File Name: Supplementary Data 2
Description: List of crosslink sites from Supplementary Data 1, but in case of multiple identifications of crosslink sites in the different replicates, only the copy of each crosslink site with higher aggregate score was retained.

File Name: Supplementary Data 3
Description: List of crosslink sites from Supplementary Data 2, but only crosslink sites with an aggregate score higher than 250 were retained.
