# Supplementary material for: The augmin complex architecture reveals structural insights into microtubule branching
Source: Nat Commun. 2022 Sep 26;13:5635. doi: 10.1038/s41467-022-33228-6 (PMC9512787; doi:10.1038/s41467-022-33228-6)
Supplement: Supplementary file 7 — Reporting Summary [file 41467_2022_33228_MOESM7_ESM.pdf]

nature research

Corresponding author(s): Stefan Pfeffer, Elmar Schiebel, Sebastian Eustermann

Last updated by author(s): Aug 25, 2022

Reporting Summary

Nature Research wishes to improve the reproducibility of the work that we publish. This form provides structure for consistency and transparency in reporting. For further information on Nature Research policies, see our Editorial Policies and the Editorial Policy Checklist.

Statistics

For all statistical analyses, confirm that the following items are present in the figure legend, table legend, main text, or Methods section.

n/a	Confirmed	
☐	☒	The exact sample size (*n*) for each experimental group/condition, given as a discrete number and unit of measurement
☐	☒	A statement on whether measurements were taken from distinct samples or whether the same sample was measured repeatedly
☒	☐	The statistical test(s) used AND whether they are one- or two-sided<br>*Only common tests should be described solely by name; describe more complex techniques in the Methods section.*
☒	☐	A description of all covariates tested
☒	☐	A description of any assumptions or corrections, such as tests of normality and adjustment for multiple comparisons
☒	☐	A full description of the statistical parameters including central tendency (e.g. means) or other basic estimates (e.g. regression coefficient) AND variation (e.g. standard deviation) or associated estimates of uncertainty (e.g. confidence intervals)
☒	☐	For null hypothesis testing, the test statistic (e.g. *F*, *t*, *r*) with confidence intervals, effect sizes, degrees of freedom and *P* value noted<br>*Give P values as exact values whenever suitable.*
☒	☐	For Bayesian analysis, information on the choice of priors and Markov chain Monte Carlo settings
☒	☐	For hierarchical and complex designs, identification of the appropriate level for tests and full reporting of outcomes
☒	☐	Estimates of effect sizes (e.g. Cohen's *d*, Pearson's *r*), indicating how they were calculated

Our web collection on statistics for biologists contains articles on many of the points above.

Software and code

Policy information about availability of computer code

Data collection	Dataset for cryo-EM was collected with EPU (version 2.6) at Krios TEM equipped with Gatan K3 camera operated by Gatan Microscopy Suite (version 3.32). Datasets for negative stain were collected at Talos L120C equipped with Ceta 16M using Thermo Fischer Scientific software EPU 2.9 . Anion exchange chromatography and size exclusion chromatography was performed at Äkta go operated by Unicorn (version 7.6). SDS page images were acquired with LAS4000IR v2.1 software. Crosslinking data were collected on Orbitrap Fusion Lumos.
Data analysis	Cryo-EM data were processed using Relion 3.1, Warp 1.0.7 , cryoSPARC 3.2 , MotionCor2 v1.0.5. and Gctf 1.06. All density map-related figures and geometric analyses were prepared/performed in UCSF Chimera 1.13.1 and UCSF ChimeraX 1.3 and segmentation in UCSF Chimera was performed by Segger 1.9.5. Model building of augmin octamer was performed in UCSF Chimera 1.13.1 and Coot 0.9.6 . Refinement and flexible fitting of the augmin TIII model was performed using the Namdinator v2.0. All model validations were done in Phenix 1.20. Analysis and vector visualisation of augmin was performed in PyMOL 2.1. Negative stain data were processed using Relion 3.1, Gctf 1.06. FSC curves for cryo-EM data were plotted in cryoSPARC 3.2. Crosslinking data of augmin were analysed in Mass Spec Studio 2.4.0.3524 and visualised using xVis 1.0 online tool. Model validation by crosslinking data was performed using Xlink Analyzer 1.1.2 beta. Model predictions were run on AlphaFold 2.2.0 with auxilary databases HMMER 3.3.2, HH-suite 3.3.0, Kalign 3.3.1. SDS page images were analyzed by Fiji (ImageJ v2.1.0/1.53c). Prism v9.1 (GraphPad Software) was used for the data representation. Microsoft Excel (v16.46.21021202) was used for data preparation.

For manuscripts utilizing custom algorithms or software that are central to the research but not yet described in published literature, software must be made available to editors and reviewers. We strongly encourage code deposition in a community repository (e.g. GitHub). See the Nature Research guidelines for submitting code & software for further information.

Data

Policy information about availability of data

All manuscripts must include a data availability statement. This statement should provide the following information, where applicable:

- Accession codes, unique identifiers, or web links for publicly available datasets
- A list of figures that have associated raw data
- A description of any restrictions on data availability

Atomic coordinates and the associated negative stain EM and cryo-EM densities have been deposited in the Protein Data Bank and the Electron Microscopy Data Bank under accession codes PDB-8AT2 [https://www.rcsb.org/structure/8AT2] /EMD-15631 [https://www.ebi.ac.uk/pdbe/entry/emdb/EMD-15631], PDB-8AT3 [https://www.rcsb.org/structure/8AT3] /EMD-15632 [https://www.ebi.ac.uk/pdbe/entry/emdb/EMD-15632], PDB-8AT4 [https://www.rcsb.org/structure/8AT4] / EMD-15633 [https://www.ebi.ac.uk/pdbe/entry/emdb/EMD-15633]. Models predicted by AlphaFold-Multimer are available at the ModelArchive with the identifiers ma-wpr7k [https://modelarchive.org/doi/10.5452/ma-wpr7k], ma-wsse3 [https://modelarchive.org/doi/10.5452/ma-wsse3], ma-w3l0m [https://modelarchive.org/doi/10.5452/ma-w3l0m], ma-8yvsa [https://modelarchive.org/doi/10.5452/ma-8yvsa]. The mass spectrometry proteomics data have been deposited in the ProteomeXchange Consortium 67 via the PRIDE 68 [http://www.proteomexchange.org/] partner repository with the dataset identifier PXD034895 [http://proteomecentral.proteomexchange.org/cgi/GetDataset?ID=PXD034895]. Source data are provided with this paper. DNA constructs generated in this study are available upon request to the corresponding authors. Published structural data used in this article: PDB-3IZ0 [https://www.rcsb.org/structure/3IZ0], PDB-3JAR [https://www.rcsb.org/structure/3JAR], PDB-6EW0 [https://www.rcsb.org/structure/6EW0].

Field-specific reporting

Please select the one below that is the best fit for your research. If you are not sure, read the appropriate sections before making your selection.

[x] Life sciences [ ] Behavioural & social sciences [ ] Ecological, evolutionary & environmental sciences

For a reference copy of the document with all sections, see nature.com/documents/nr-reporting-summary-flat.pdf

Life sciences study design

All studies must disclose on these points even when the disclosure is negative.

Sample size	For cryo-EM data, no statistical method was chosen to determine the sample size. The number of micrographs was chosen to obtain a number of particles sufficient to reconstruct a detailed 3D density of the augmin TIII tetramer. Collected cryo-EM data contained 12,615 images of augmin TIII tetramer, corresponding to a two-day microscopy session with parameters specified in the method section. Initial number of picked particles was 1,060,446. After two consecutive rounds of 2D classification, we reached the final dataset of 82,776 particles that were used for homogeneous refinement in cryoSPARC. The final reconstructions reached resolution that was estimated by Gold Standard FSC method. AlphaFold predictions produced 5x5 models per each construct. For negative stain EM data, no statistical method was chosen to determine the sample size. The number of micrographs was chosen to obtain a number of particles sufficient for in-depth 2D and 3D class averaging. For augmin particles at the given resolution this was around 500 micrographs. 504 images for augmin TIII were acquired. 412,188 particles were picked initially and submitted to 5 consecutive rounds of 2D classification, after which 13,594 particles were selected for one round of 3D classification after which 11,897 particles were selected for 3D refinement. For augmin octamer, 583 images were acquired, 80,837 particles were picked initially and submitted to 2 consecutive rounds of 2D classification, after which 56,021 particles were selected for one run of 3D classfication and a final 2D classification. After 3D classfication, two individual classes (11,969 particles and 10,658 particles) were used for separate 3D refinements and 2D classification. No statistical method was used to determine the sample size of protein expressions, protein purifications and BS3 crosslinking experiment for crosslinking mass-spec analysis. The sample size was sufficient for negative stain EM, cryo-EM and crosslinking mass-spec analysis.
Data exclusions	All collected negative stain EM and cryo-EM images were used for a particle picking. Cryo-EM particle selection was performed in two 2D classification rounds and criterion was based on the shape and quality of resulting 2D class averages. The class averages representing different views were selected for homogeneous refinement in order to minimise effect of preferential orientation.<br>Negative stain EM particle selection was performed in several 2D classification rounds and criterion was based on the shape of the class averages.
Replication	Protein expression and purification were done in at least 3 replicates. Negative stain EM data acquisition and subsequent SDS page analysis of samples subjected to negative stain EM were performed once for each dataset (0 repetitions). Cryo-EM data were acquired in one session on one grid (0 repetitions). For cryo-EM and negative stain EM analysis, the final 2D classes and 3D densities are averages of thousands of particles and thus repetitions of the experiments were not necessary. Alphafold predictions produce five predicted models per five random seeds as default settings for a prediction. Crosslinking data were collected in three technical replicates, which were all successful.
Randomization	Positions for automatic image acquisition in cryo-EM and negative stain EM were selected based on the presence of the particles. The particles on the images were selected by a computer program (Relion 3.1 (negative stain EM) or BoxNet in Warp (cryo-EM)) in an unsupervised manner. Other experiments are not related to randomization as randomization was not applicable to other experiments like cloning, protein expression and protein purification.

Blinding	Cryo-EM, negative stain EM analysis and crosslinking data analysis were not blinded because they are performed computationally. AlphaFold prediction is performed computationally thus is not blinded. For other experiments like cloning, protein expression and protein purification blinding was not perfomed because comparisons amoung samples in these experiments were not the scope of this study and therefore blinding could not affect the results.

Reporting for specific materials, systems and methods

We require information from authors about some types of materials, experimental systems and methods used in many studies. Here, indicate whether each material, system or method listed is relevant to your study. If you are not sure if a list item applies to your research, read the appropriate section before selecting a response.

Materials & experimental systems

n/a	Involved in the study	
☒	☐	Antibodies
☐	☒	Eukaryotic cell lines
☒	☐	Palaeontology and archaeology
☒	☐	Animals and other organisms
☒	☐	Human research participants
☒	☐	Clinical data
☒	☐	Dual use research of concern

Methods

n/a	Involved in the study	
☒	☐	ChIP-seq
☒	☐	Flow cytometry
☒	☐	MRI-based neuroimaging

Eukaryotic cell lines

Policy information about cell lines

Cell line source(s)	SF21 cells were obtained from EMBL Protein Expression and Purification Core Facility, Heidelberg Germany.<br>High Five cell line was purchased from Invitrogen (BTI-TN-5B1-4, cat no. B855-02).
Authentication	The cell lines were not authenticated.
Mycoplasma contamination	Mycoplasma test was negative for all cell lines.
Commonly misidentified lines<br>(See ICLAC register)	No misidentified cell lines in this study.
